# Molecular Signature of Cancer at Gene Level or Pathway Level? Case Studies of Colorectal Cancer and Prostate Cancer Microarray Data

**DOI:** 10.1155/2013/909525

**Published:** 2013-01-16

**Authors:** Jiajia Chen, Ying Wang, Bairong Shen, Daqing Zhang

**Affiliations:** ^1^Center for Systems Biology, Soochow University, Jiangsu, Suzhou 215006, China; ^2^Department of Chemistry and Biological Engineering, Suzhou University of Science and Technology, Jiangsu, Suzhou 215011, China; ^3^Laboratory of Gene and Viral Therapy, Eastern Hepatobiliary Surgical Hospital, Second Military Medical University, Shanghai 200438, China

## Abstract

With recent advances in microarray technology, there has been a flourish in genome-scale identification of molecular signatures for cancer. However, the differentially expressed genes obtained by different laboratories are highly divergent. The present discrepancy at gene level indicates a need for a novel strategy to obtain more robust signatures for cancer. In this paper we hypothesize that (1) the expression signatures of different cancer microarray datasets are more similar at pathway level than at gene level; (2) the comparability of the cancer molecular mechanisms of different individuals is related to their genetic similarities. In support of the hypotheses, we summarized theoretical and experimental evidences, and conducted case studies on colorectal and prostate cancer microarray datasets. Based on the above assumption, we propose that reliable cancer signatures should be investigated in the context of biological pathways, within a cohort of genetically homogeneous population. It is hoped that the hypotheses can guide future research in cancer mechanism and signature discovery.

## 1. Introduction

Microarray technology has evolved rapidly in the past several years as a powerful tool for large-scale gene expression profiling [1]. By monitoring changes in gene expression patterns, microarray technology is widely utilized in search of molecular signatures for many medical conditions including cancer. However, evidence is mounting that differentially expressed gene (DEG) lists detected from different studies for the same disease are often inconsistent [2, 3]. One might attribute the inconsistency to the variation in microarray platforms, experimental samples, normalization and analysis methods, and inherent biological uncertainty. Yet this discordance remains even in technical replicate tests using identical samples as in the case of Ein-Dor et al. [4]. Therefore, signature identification at the level of differential genes has been challenged about its robustness and reliability. In light of the inconsistency between DEG lists obtained from different datasets, we propose herein two hypotheses: (1) the expression signatures of different cancer microarray datasets are more similar at pathway level than at gene level; (2) the comparability of the cancer molecular mechanisms of different individuals is related to their genetic similarities. The hypotheses are subsequently verified by case studies of colorectal cancer and prostate cancer microarray datasets, respectively. Hopefully, the hypotheses would explain the inconsistency of the DEG lists derived from multiple experiments and provide novel methods for discovering robust and specific biomarkers of cancer.

## 2. Materials and Methods

### 2.1. Data Collection

We collected 5 gene expression profiling datasets on colorectal cancer and 10 datasets on prostate cancer from public gene expression data repositories, for example, Gene Expression Omnibus (GEO), Oncomine [5] and Supplementary Materials from published literatures. The detailed information of the datasets was summarized in Table 1 for colorectal cancer and Supplementary Table  1 (see Supplementary Material available online at http://dx.doi.org/10.1155/2013/909525) for prostate cancer. These data were collected from two types of platforms, that is, cDNA two-channel arrays and Affymetrix microarray platforms including Human 6800 Affy gene chips, HG-U95A and HG-U133 series. Each dataset was named after the first author of the original literature. Only profiles of normal and cancer tissues were extracted for further analysis.

### 2.2. Preprocessing of Raw Data

The images of the cDNA array were processed using GenePix Pro 5.0.1.24 software. Background correction was performed by subtracting the median background intensities from the median foreground intensities of all spots in both channels. The raw datasets measured with Affymetrix chips were analysed via MAS5.0 algorithm in R platform. To eliminate the systematic error from heterogeneous datasets before the identification of signatures, we performed Locally Weighted Scatter Plot Smoothing (LOWESS) method for within-chip normalization of cDNA array's dataset and Median Absolute Deviation (MAD) method for between-chip normalization of all datasets. In addition, data was filtered to eliminate bad spots, and the filter criterion was defined as 60% absence across all of the samples. All of the data of preprocessing procedures were performed in R programming environment. 

### 2.3. Determination of the Differentially Expressed Outlier Genes

Cancer Outlier Profile Analysis (COPA) method was performed for detecting genes that were differentially expressed between cancer and normal samples. We used COPA package by MacDonald and Ghosh [6] in R platform. According to the COPA package guidelines, the data was centered and scaled on a rowwise basis using median average difference. The rows of microarray expression data matrix were genes, and the columns were samples. The COPA function calculates a “COPA” score from a set of microarrays. As a preliminary step the function used a percentile for pre-filtering the data. The number of outlier samples for each gene was calculated, and all genes with a number of outlier samples less than the percentile (default 95th) were removed from further consideration. A threshold cutoff for “outlier” status was set as 1.7 and applied to all genes. 

### 2.4. Functional Enrichment of Outlier Genes

The significant outlier genes were subsequently mapped to functional databases, for example, GSEA [7], KEGG [8], and GeneGO (GeneGO, Inc.) for the pathway enrichment analysis. GSEA analysis and KEGG pathway analysis were performed using Gene Set Enrichment Analysis (GSEA) tool [7] and Onto-Express [9, 10], respectively. GSEA tool used a collection of gene sets from molecular signatures database (MSigDB), which was divided into five major collections. In our work, we used C2 curated gene sets. Enriched GeneGO pathways were detected by MetaCore (GeneGO, Inc) [11] software. *P*-value was used to evaluate the statistical significance of each candidate pathway. In MetaCore, the statistics significance (*P*-value) was calculated by using hypergeometric distribution. False Discovery Rate (FDR) adjustment was applied for multiple test correction.

### 2.5. Pairwise Overlapping Comparison at Gene/Pathway Level

The overlapping percentage between two datasets is calculated as follows:

(1) $$\begin{array}{c} \text{Overlapping}\;\text{percentage}=\frac{m}{n_{1}+n_{2}-m}\times 100\%, \end{array}$$

where *n*_1_ is the number of all the data in dataset 1, *n*_2_ is the number of all the data in dataset 2, and *m* is the number of overlapping data between two datasets. 

## 3. Results

### 3.1. Outlier Detection Using Novel Statistic Method

Table 1 listed the statistical methods for identifying differentially expressed genes by the original articles. Most of the prevailing analytical methods like *t*-test, SAM, and *z*-statistic considered the average value of gene intensities in the cancer samples. These statistical methods, however, would fail to find “outlier genes” which are only involved in subsets of the cancer samples. Despite their scarcity, outlier genes are nontrivial and may present a hallmark of potential oncogenes. These conventional methods are not suitable for detecting such subset-specific oncogene expression profiles as proposed by Tomlins et al. [12] and Lian [13]. Through applications to public cancer microarray datasets in our previous study [14], we have demonstrated that some newly developed statistics showed superior performance than traditional *t*-statistics in outlier detection. We herein applied Cancer Outlier Profile Analysis (COPA), a novel significant genes analysis method proposed by Tomlins et al. [12], to meta-analyze multiple cancer datasets. 

### 3.2. Signatures Are More Similar at Pathway Level across Multiple Colorectal Cancer Datasets

In order to verify our first hypothesis, we performed meta-analysis of 5 colorectal cancer gene expression profiling datasets from independent laboratories [15–19]. 

After COPA analysis, we identified 3258 genes differentially expressed between normal colorectal and colorectal tumor samples. The searches in the Entrez PubMed database showed that only 450 out of 3258 (13.8%) identified genes by COPA method were associated with colorectal cancer.

The number of overexpressed genes was obviously discrepant across all groups because of the different samples, arrays, and platforms. To decrease the discrepancy, we tried to understand the cancer molecular mechanism at systems biological level. We then mapped the DEGs identified by COPA using Gene Set Enrichment Analysis (GSEA) and MetaCore software for pathway enrichment analysis, respectively. Totally we found 262 enriched pathways in GeneGO's database with a *P* value threshold of 0.05; the detailed list of the pathways are provided in Supplementary Table  2. In addition, we performed the gene sets enrichment analysis in GSEA by using C2 curated file, which includes 1892 gene sets/pathway annotation. 111 outlier gene sets with NOM *P*-value <0.05 and FDR < 0.05 were also found and listed in Supplementary Table  3. The numbers of significant GeneGO pathways or GSEA gene sets enriched by the differentially expressed gene for 5 colorectal cancer datasets were listed in Table 2.

**Figure 1:**
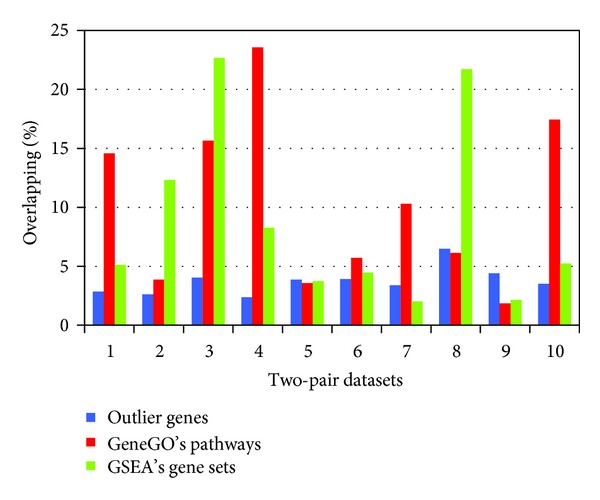
Pairwise overlapping percentage of 5 datasets among differentially expressed genes, enriched gene sets in GSEA, and enriched pathways in GeneGO database. The *x*-axis represented all the two-pair combination of 5 datasets. The *y*-axis represented the overlapping percentage.

We performed pairwise comparison between 5 datasets in terms of DEGs, GSEA's enriched gene sets, and GeneGO's enriched pathways, respectively. For 5 different datasets, 10 pairs of datasets are available for comparison.  Figure 1 showed the pairwise overlapping percentage at different observation levels. A significantly higher overlap at pathway level than at gene level is observed with 70% of the dataset pairs by GeneGO and 60% of the dataset pairs by GSEA. This observation supports our first hypothesis that the overlapping percentage at the pathway level is higher than that at the gene level. 

Moreover, we found 4 GeneGO pathways that were shared by 4 datasets. These pathways were considered to be most overlapped and listed in Table 3. Among them, ECM remodeling, chemokines, and adhesion pathways, belonging to cell adhesion category, were previously reported to play a role in colorectal cancer. The other two pathways, integrin outside-in signalling pathway and L-selenoamino acids incorporation in proteins during translation pathway, have not been reported as colorectal cancer associated pathways. The network objects in both of the pathways, however, have been widely reported in colorectal cancer. Integrins are heterodimeric adhesion receptors, and most of them recognize ECM proteins. A major function of integrin signaling is to link ECM proteins to intracellular actin filaments via interactions of integrins with actin-binding proteins. Therefore, the correlation between integrin signaling and ECM pathway may play an active role in colorectal cancer. We infer that these two pathways might be putative novel colorectal cancer related pathways which could provide crucial guidance for biological scientists. Their roles in colorectal cancer need further experimental validation in the future.

We performed paired *t*-test to decide whether the different overlapping percentages observed between different levels are significant. The *P*-values for the difference between outlier genes and GeneGO's enriched pathways were 0.01354 by paired *t*-test and 0.02441 by Wilcoxon test. The *P*-values for the difference between outlier genes and GSEA gene sets were 0.028 by paired *t*-test and 0.08 by Wilcoxon test, respectively. The *P*-values indicate that the overlapping percentages at gene set or pathway level are significantly higher than that at individual gene level. We thus came to the conclusion that the expression signatures of independent datasets at higher functional level are significantly more consistent than that at gene level.

### 3.3. The Prostate Cancer Outlier Gene Enriched Pathways Show a Regional Distribution Feature

In support of the second hypothesis, we performed a regional analysis of 10 publicly available prostate cancer gene-expression datasets from different locations [20–28].

We first conducted KEGG and GeneGO pathway enrichment analysis on these datasets, followed by a pairwise comparison of pathway overlapping percentage among them. Only the significantly enriched pathways with previous evidence of prostate cancer association were adopted for the comparison. Text mining was performed to make sure that there was at least one published paper describing the function of these pathways in prostate cancer. 

**Figure 2:**
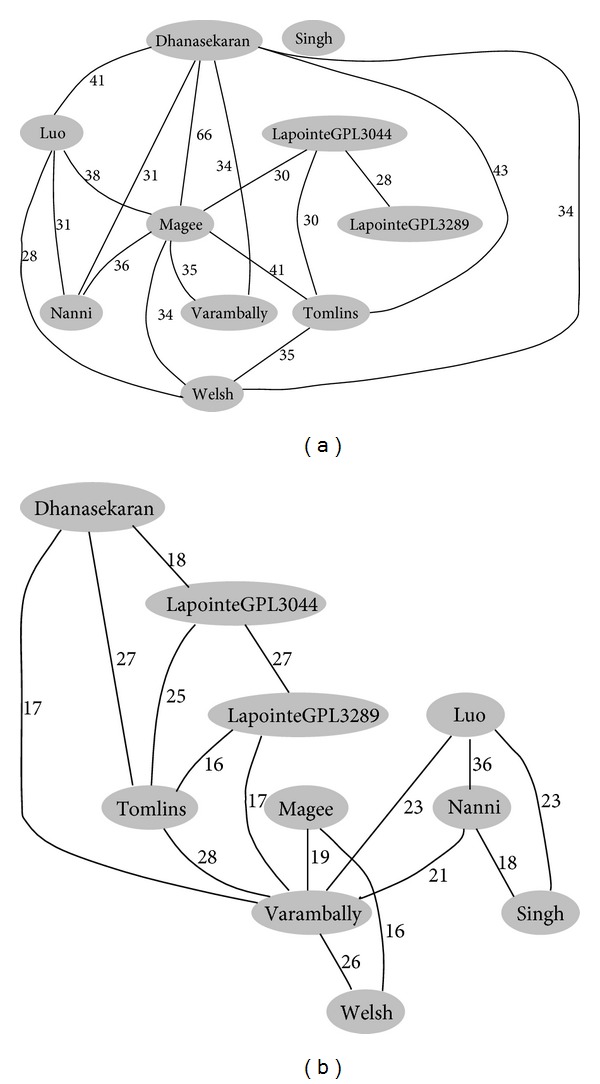
A simple network that associates datasets according to their similarity distances. The distances were calculated based on the overlapping percentage of the enriched pathways identified by (a) GeneGO and (b) KEGG. The lines between two datasets mean that their overlapping is more than two-thirds of the all. Each circle represented a dataset, and the overlapping percentage was shown on the lines.

Based on pathway overlapping analysis, we calculated the distance matrices between these datasets and generated a network to display their association. Five common distances, that is, Euclidean distance, Pearson correlational distance, Manhattan distance, Kendall's tau correlational distance, and Hamming distance were used to measure the similarity of these datasets. Based on these distances, a network graph was generated to display the association of these datasets. Figures 2(a) and 2(b) illustrate the association of the datasets based on GeneGO pathways and KEGG pathways, respectively. 

Figure 2 revealed an essential regional distribution feature of significant pathways across multiple datasets. It is obvious from the graph that the distance between two Lapointed [29] datasets is the closest among all the datasets. Datasets by Dhanasekaran et al. [20], Tomlins et al. [25], and Magee et al. [23] feature a high pathway overlap which could be reflected by distances, indicating their similarities. The datasets from Singh et al. [26], Luo et al. [22], Welsh et al. [24], and Nanni et al. [27] diverge less from each other than those from the other six datasets.

We then investigated the regional sources of the tissue specimens for each dataset, as listed in Table 4. Samples of Dhanasekaran et al. [20] and Tomlins et al. [25] were obtained from the same place; those of Magee et al. [23] were close to them. Samples of Singh et al. [26], Welsh et al. [24] and Luo et al. [22, 30] came from adjacent states in America. Although the samples of Lapointe et al. [21] were not given a specific location, the author informed us their two experiment datasets were taken from patients of the same population. Apparently, there is obvious concordance between dataset similarity and sample source distribution. 

**Figure 3:**
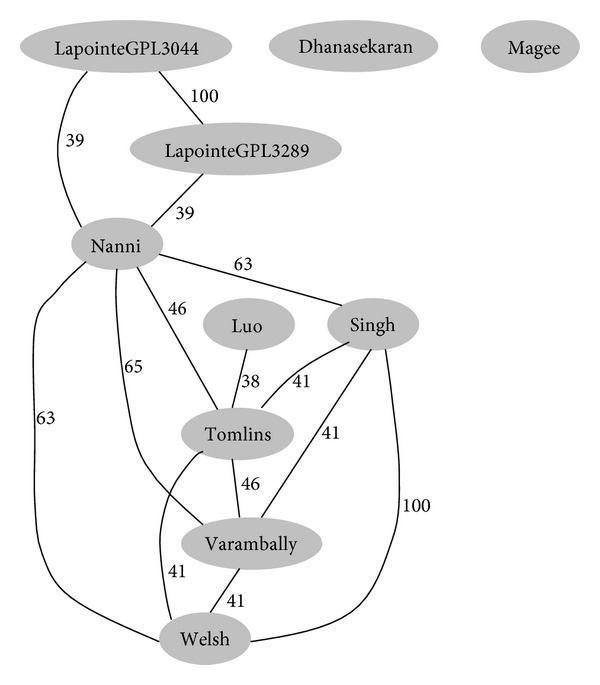
A simple network that associates datasets according to the similarity in microarray platforms. The distances represent the overlapping proportion of the probes used in different platforms.

Considering the influence by different microarray platforms, we compared the total unique genes of each dataset in order to testify that the significant pathway distribution feature is caused by different data sources rather than different experimental platforms. As implied in Figure 3, the similarities of the experimental platforms, here the overlapping proportion of the nonredundant probes used in different platforms, are not correlated to the regional distribution. Therefore, the regional distribution of cancer signature at pathway level is independent of the experimental platforms. 

## 4. Discussion

### 4.1. Comparison of DEGs between Different Experiments Revealed Little Overlap

The application of DNA microarrays for the investigation of cancer has led to numerous microarray studies that examined the same clinical conditions. Nevertheless, experiments from different groups have given dissimilar results when DEG lists are directly compared. The disparity was demonstrated in this study, where a meta-analysis of 5 colorectal cancer microarray expression datasets from 4 independent laboratories was performed. We calculated the pairwise overlapping proportion of DEGs between any two datasets, only to find that the overlap between the two lists was disappointingly small (~5%).

Such inconsistency has been observed in gene expression profiling of various types of cancer. For example, in two prominent studies that aimed to predict survival of breast cancer patients [31, 32], both groups claimed to have generated gene lists with predictive power, but only 17 genes appeared on both lists. In another attempt to predict the 5-year metastasis of breast cancer, van't Veer et al. [31] and Wang et al. [33] reported a list of gene sets with good prediction performance, respectively. But the predictive success of their studies was frustrated by the fact that the sets of metastasis-related genes identified by these two independent studies had only 3 overlapping genes. More recently our colleagues [3] meta-analyzed 10 independent microarray datasets associated with prostate cancer, but the resulting set of DEGs had only ~20% overlap between each datasets.

The most straightforward explanation of this lack of agreement is the variation in microarray platforms, experimental samples, normalization, and analysis methods. The open question is, however, whether the inconsistency can be attributed only to these trivial reasons? 

To address the issue, Ein-Dor et al. [4] sought to remove all the technical differences mentioned above by analyzing a single breast cancer dataset [31] with a single method. By randomly generating training datasets, they demonstrated that the same analysis could have obtained many equally predictive gene lists and that two such lists share, typically, only a small number of genes. This finding indicates that low consistency occurs even in technical replicate tests using identical samples. The reason for this inconsistency or instability would be that (1) the number of DEGs is large whereas the number of samples is limited; (2) the resulting set of DEGs fluctuates according to the subset of patients used for gene selection.

### 4.2. Identifying Robust Molecular Signatures at Functional Modules Level or Pathway Level

In this study we evaluated the consistency of signatures across 5 colorectal cancer datasets produced by different platforms. Although the DEG lists selected had only ~5% overlaps, their enriched pathways were still consistent. Consistency analysis at different levels provides solid evidence that cancer signatures at pathway level diminish the discrepancies observed in direct comparisons of DEGs and are more consistent across multiple datasets than at gene level.

As the understanding of tumor biology deepens, it is well recognized that carcinogenesis is characterized with coordinated molecular changes. Functionally correlated genes often display coordinated expression to accomplish their roles; one would therefore expect that the inconsistent DEG lists across independent experiments are functionally more consistent. In other words, the discrepancies of DEGs would be less pronounced when they are mapped to functional groups or biological pathways.

Following this line, some previous studies have shifted their focus from individual genes to the biologically related groups of genes in the analysis of cancer microarray data. For example, in order to investigate the robustness of biological themes, Hosack et al. [34] applied the Expression Analysis Systematic Explorer (EASE) to determine the biological theme for DEG lists generated by various gene selection methods. Their research provided strong evidence that biological themes are stable to varying methods of gene selection. Zhu et al. [35] developed a novel tool for identifying cancer signatures at functional modules level. Its applications to two cancer types demonstrated that the functional modules enjoy explicit relevance to cancer biology. Recently, Yang et al. [36] proposed semantic similarity measure for DEG lists detected under varied statistical thresholds and from different studies. They reported that gene lists could be functionally consistent according to their semantic similarity. In addition, Gorlov et al. [37] conducted functional annotation analysis of the prostate cancer genes identified by two different methods. They observed a considerable overlap between biological functions identified by varied methods.

In recent years, pathway analysis has received a great deal of attention in the study of cancer microarray data [7, 34]. Pathway analysis typically correlates the identified DEGs with predefined pathway databases. It is reported that pathway analysis applied to differential gene lists detected under varied statistical methods yielded common results [38]. This discovery was validated in our previous study by Wang et al. [3], who evaluated the consistency of signature across 10 prostate cancer datasets produced by different platforms. Although the datasets share disappointingly few DEGs, their DEG-enriched pathways were still consistent. 

### 4.3. Searching for Common Signatures among a Cohort of Genetic Homogeneous Population

As for the second hypothesis we assume that the individuals bearing similar genetic/environmental factors tend to share more common pathways. However, the information on the genetic/environmental characteristics of the patient samples is generally lacking. We believe it should be statistically reasonable to take the geographical location of the sample resources as the measurement of the similarities of their genetic/environmental factors. According to the similarity of outlier enriched pathways found by GeneGO and KEGG, we are able to classify 10 different prostate cancer related datasets into several groups. The datasets from same or adjacent geographical locations tend to reside within the same group. In other words, we observed an essential regional distribution feature of significant pathways across multiple datasets. In this sense molecular signatures from the geographically adjacent tissue specimens would be more consistent than those generated from geographically isolated samples. This observation is basically in accordance with our hypothesis that the comparability of the cancer molecular mechanisms of different individuals is related to their genetic similarities.

Cancer represents a heterogeneous disease, which reflects the interaction of a myriad of etiological and genetic contributions [39]. Therefore the gene expression profiles of cancer patients are diverse, depending on factors such as genetic information, environment effect, and personal behaviors. The role of genetic and environmental factors in modulating gene expression variation in humans has been extensively investigated. Most of the previous studies on cancer microarray profiling, however, ignored the interindividual variation in gene expression. It is likely that differences in expression that appear to be related with the disease may in fact represent random genetic variation. This situation will further introduce false discoveries and reduce the overall reproducibility of DEG detection. This concern was mentioned by Michiels et al. [40], who investigated the stability of seven published datasets to predict prognosis of cancer patients. It was observed that the predictive gene lists reported by the various groups were highly unstable and depended strongly on the subset of samples chosen for training.

It is assessed that, to achieve a typical overlap of 50% between two predictive lists of genes, the expression profiles of several thousands of patients would be needed [41]. Unfortunately, obtaining such a large number of samples is currently impractical due to limited tissue availability and financial constraints. A more practical approach would be to search for common signatures among a genetically homogeneous human population other than those among a mixed population. Although different individuals may have different regulatory mechanisms and discrepant cancer associated pathways, we assume that the individuals bearing similar genetic and environmental factors tend to share more common pathways. 

Thus it would be reasonable to group patients into well-defined small subgroups on the basis of each person's unique genetic and environmental information. In this way, the individual difference of cancer mechanism is accounted when we analyze cancer expression data from different resources. This kind of investigation will help to find population-specific cancer pathways and facilitate personalized medicine.

## 5. Conclusions

Based on previous observations, we proposed herein two novel points of view for the cancer signatures identification. The pathway-based approach suggested in this paper would hopefully improve the comparability of different microarray datasets and, therefore, may lead to more valid and reliable biological interpretation of microarray results. Moreover, the generation of the population-specific cancer signatures would help to deliver effective therapy to patients most likely to benefit from such treatment and enable “personalized medicine.” With increasing amount of cancer datasets available, the challenge in the future is to collect more cancer datasets from independent populations to prove our hypotheses.

## Supplementary Material

Supplementary Table 1: The detailed information of the prostate cancer gene expression data sets used in the meta-analysis.
Supplementary Table 2: The detailed list of the 262 GeneGO pathways that are enriched with DEGs (p-value < 0.05)
Supplementary Table 3: The detailed list of the 111 GSEA gene sets that are enriched with DEGs (NOM p-value < 0.05 and FDR q-value < 0.05)

## Figures and Tables

**Table 1:** Colorectal cancer gene expression datasets used in the meta-analysis.

Dataset	Platform	Total genes	Total samples	Experimental design		Statistical method
				Normal	Tumor
Hong	HGU133	54675	22	10	12	*t*-test
Sabates-Bellver	HGU133	54675	64	32	32	Mann-Whitney test
Galamb1	HGU133	54675	30	11	19	SAM
Galamb2	HGU133	54675	38	8	30	PAM
Graudens	cDNA	23232	30	12	18	*z*-statistics

SAM: significance analysis of microarrays; PAM: prediction analysis of microarrays.

**Table 2:** The number of pathway/gene sets enriched by differentially expressed gene for five colorectal cancer datasets.

Dataset	Number of enriched pathways in GeneGO	Number of enriched gene sets in GSEA
Hong	71	154
Sabates-Bellver	50	303
Galamb1	78	91
Galamb2	36	128
Graudens	149	172

**Table 3:** The top 4 most overlapped GeneGO's pathways shared by 4 datasets.

GeneGO ontology	Pathway name	Pubmed citation count
Translation	(L)-selenoamino acids incorporation in proteins during translation	0
Cytoskeleton remodeling	Integrin outside-in signaling	0
Cell adhesion	ECM remodeling	64
Cell adhesion	Chemokines and adhesion	1117

**Table 4:** Tissue specimen sources of each prostate cancer expression dataset.

Datasets	Tissue specimens sources	Locations
Dhanasekaran	University of Michigan Specialized Program of Research Excellence in Prostate Cancer (SPORE) tumor bank	America, Michigan (MI)
Lapointe	Stanford University; Karolinska Institute; Johns Hopkins University	America, California (CA); Sweden, just outside Stockholm; America, Maryland (MD);
Tomlins	University of Michigan	America, Michigan (MI)
Luo	Johns Hopkins Hospital	America, Maryland (MD)
Magee	Washington University School of Medicine; University of Washington Medical Center	America, Missouri (MO); America, Washington (WA);
Welsh	University of Virginia (UVA)	America, Virginia (VA)
Varambally	University of Michigan Prostate Cancer Specialized Program of Research Excellence (SPORE) Tissue Core	America, Michigan (MI)
Singh	Brigham and Women's Hospital	America, Massachusetts (MA)
Nanni	Regina Elena Cancer Institute	Italy, Rome
